# Knockout of the Complex III subunit *Uqcrh* causes bioenergetic impairment and cardiac contractile dysfunction

**DOI:** 10.1007/s00335-022-09973-w

**Published:** 2022-12-24

**Authors:** Nadine Spielmann, Christina Schenkl, Tímea Komlódi, Patricia da Silva-Buttkus, Estelle Heyne, Jana Rohde, Oana V. Amarie, Birgit Rathkolb, Erich Gnaiger, Torsten Doenst, Helmut Fuchs, Valérie Gailus-Durner, Martin Hrabě de Angelis, Marten Szibor

**Affiliations:** 1 Institute of Experimental Genetics, German Mouse Clinic, Helmholtz Center Munich, German Research Center for Environmental Health, Ingolstädter Landstr. 1, 85764 Neuherberg, Germany; 2 Department of Cardiothoracic Surgery, Center for Sepsis Control and Care (CSCC), Jena University Hospital, Friedrich Schiller University of Jena, Am Klinikum 1, 07747 Jena, Germany; 3 Oroboros Instruments, Schöpfstr. 18, 6020 Innsbruck, Austria; 4 Department of Biochemistry and Molecular Biology, Semmelweis University, Tuzoltostreet 37-47, 1094 Budapest, Hungary; 5 BioMediTech & Tampere University Hospital, Faculty of Medicine and Health Technology, Tampere University, Arvo Ylpön Katu 34, 33520 Tampere, Finland; 6 Institute of Molecular Animal Breeding and Biotechnology, Gene Center, Ludwig-Maximilians-Universität München, Feodor-Lynen Str. 25, 81377 Munich, Germany; 7 Member of German Center for Diabetes Research (DZD), Ingolstädter Landstr. 1, 85764 Neuherberg, Germany; 8 Chair of Experimental Genetics, School of Life Science Weihenstephan, Technische Universität München, 85354 Freising, Germany

## Abstract

Ubiquinol cytochrome *c* reductase hinge protein (UQCRH) is required for the electron transfer between cytochrome *c*_1_ and *c* of the mitochondrial cytochrome *bc*_1_ Complex (CIII). A two-exon deletion in the human *UQCRH* gene has recently been identified as the cause for a rare familial mitochondrial disorder. Deletion of the corresponding gene in the mouse (*Uqcrh*-KO) resulted in striking biochemical and clinical similarities including impairment of CIII, failure to thrive, elevated blood glucose levels, and early death. Here, we set out to test how global ablation of the murine *Uqcrh* affects cardiac morphology and contractility, and bioenergetics. Hearts from *Uqcrh*-KO mutant mice appeared macroscopically considerably smaller compared to wildtype littermate controls despite similar geometries as confirmed by transthoracic echocardiography (TTE). Relating TTE-assessed heart to body mass revealed the development of subtle cardiac enlargement, but histopathological analysis showed no excess collagen deposition. Nonetheless, *Uqcrh*-KO hearts developed pronounced contractile dysfunction. To assess mitochondrial functions, we used the high-resolution respirometer NextGen-O2k allowing measurement of mitochondrial respiratory capacity through the electron transfer system (ETS) simultaneously with the redox state of ETS-reactive coenzyme Q (Q), or production of reactive oxygen species (ROS). Compared to wildtype littermate controls, we found decreased mitochondrial respiratory capacity and more reduced Q in *Uqcrh*-KO, indicative for an impaired ETS. Yet, mitochondrial ROS production was not generally increased. Taken together, our data suggest that *Uqcrh*-KO leads to cardiac contractile dysfunction at 9 weeks of age, which is associated with impaired bioenergetics but not with mitochondrial ROS production.

**Graphical abstract:**

Global ablation of the *Uqcrh* gene results in functional impairment of CIII associated with metabolic dysfunction and postnatal developmental arrest immediately after weaning from the mother. *Uqcrh*-KO mice show dramatically elevated blood glucose levels and decreased ability of isolated cardiac mitochondria to consume oxygen (O_2_). Impaired development (failure to thrive) after weaning manifests as a deficiency in the gain of body mass and growth of internal organ including the heart. The relative heart mass seemingly increases when organ mass calculated from transthoracic echocardiography (TTE) is normalized to body mass. Notably, the heart shows no signs of collagen deposition, yet does develop a contractile dysfunction reflected by a decrease in ejection fraction and fractional shortening. 
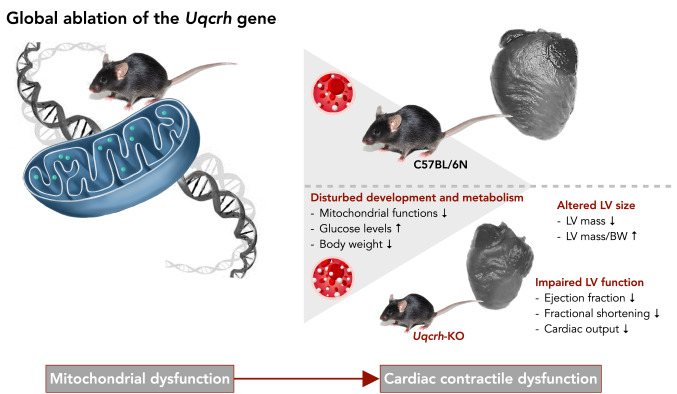

**Supplementary Information:**

The online version contains supplementary material available at 10.1007/s00335-022-09973-w.

## Background

The adult mammalian heart relies almost exclusively on oxidative phosphorylation (OXPHOS) for ATP production, a process facilitated by the mitochondrial electron transfer system (ETS). Functioning of the ETS, however, is also a vital necessity for tasks beyond ATP production, such as fatty acid, heme, and iron-sulfur cluster biosynthesis, ion homeostasis, and thermogenesis (Zhou and Tian 2018). It is, therefore, not surprising that mitochondrial fitness and cardiac contractility are mutually interconnected and that mitochondria, which account for up to 30% of cardiomyocyte volume across species (Schaper et al. 1985; Barth et al. 1992), are therapeutic targets to improve contractility in the failing mammalian heart (Mailloux 2016; Brown et al. 2017).

To warrant undisturbed functioning of the ETS, the healthy heart exhibits extraordinary metabolic flexibility, i.e., cardiomyocytes can dynamically switch between different respiratory fuel substrates, whereas metabolic inflexibility is known to account for the development of contractile malfunction and eventually heart failure (Lopaschuk et al. 2010; Lemieux et al. 2011; Muoio 2014; Bertero and Maack 2018). Under physiological conditions, fatty acid utilization by beta-oxidation prevails over glucose utilization by glycolysis, while the latter is upregulated in the failing heart (Stanley et al. 2005; Lopaschuk et al. 2010; Bertero and Maack 2018). To adapt cellular and organ functions to ever-changing demands and metabolism, mitochondria have evolved into signaling hubs that integrate metabolic signals (Chandel 2015; Martínez-Reyes and Chandel 2020; Shen et al. 2022). Mitochondria do not only adapt to substrate availability, but also communicate their metabolic state and thus mediate adaptive organ remodeling processes and stress responses (Dogan et al. 2018; Szibor et al. 2020b). In case of ETS impairment caused by pharmacological intervention or genetic defects, production of reactive oxygen species (ROS) and redox imbalance are frequently observed consequences. Such mitochondrial signaling events can be sensed by a cell (Robb et al. 2018; Dogan et al. 2018; Szibor et al. 2020a, b), and eventually lead to cardiac contractile malfunction (Rajendran et al. 2019; Dhandapani et al. 2019; Szibor et al. 2020b).

The ETS consists of several high-molecular weight respiratory Complexes, including CI-CIV. These ETS complexes jointly couple redox reactions with oxygen (O_2_) as the terminal electron acceptor to the generation of an electrochemical potential, which then is the driving force for ATP production at the F_1_F_O_-ATP synthase (Signes and Fernandez-Vizarra 2018). The functioning of CIII appears to be of particular pathogenetic importance, as we have shown that a mutation in BCS1L, a chaperone protein involved in the assembly of CIII (Fernández-Vizarra and Zeviani 2015), underlies the development of fatal cardiomyopathy (Rajendran et al. 2019). Here, we set out to test the consequences of global ablation of another CIII-related protein on cardiac contractile function, the ubiquinol cytochrome *c* reductase hinge protein (UQCRH), a regulator of electron transfer between cytochrome *c* and *c* of CIII (Kim and King 1983; Mukai et al. 1985; Kim et al. 1987; Ohta et al. 1987; Park et al. 2017). A two-exon deletion in *UQCRH* has recently been identified in two pediatric patients who developed a clinical condition characterized by recurrent episodes of severe ketoacidosis, excess blood ammonia, hypoglycemia, and signs of encephalopathy concomitant with impaired function of CIII (Vidali et al. 2021). Although UQCRH is broadly expressed across tissues, its function seems to be particularly important for organs with high-energy metabolism (Modena et al. 2003). However, an association between *UQCRH* deletion and cardiac function has not yet been investigated. Here, we took advantage of a recently generated and characterized mouse model with corresponding two-exon deletion (*Uqcrh*-KO) (Vidali et al. 2021) to explore the relationship between mitochondrial metabolism and cardiac contractile function in greater detail. The *Uqcrh*-KO mouse model largely replicates the human condition and most notably shows an impaired activity of CIII, hyperglycemia, and premature death at the age of approximately 12 weeks. We performed transthoracic echocardiography (TTE) in consecutive measurements at 6, 7, 8, and 9 weeks of age and analyzed isolated heart mitochondria at 12 weeks of age using the Oroboros high-resolution respirometer NextGen-O2k that gives in-depth information on mitochondrial respiratory capacity, ROS production, and the ETS-reactive Q redox state (Komlódi et al. 2021a).

## Results

### *Uqcrh*-KO mice show a failure to thrive and metabolic disturbance

*Uqcrh*-KO mice were generated by a two-exon deletion and show clinical signs with striking similarities, albeit a more severe phenotype compared with patients diagnosed with the corresponding deletion (Vidali et al. 2021). As a result of the mutation, both humans and mice show marked impairment of CIII activity. *Uqcrh*-KO mice were born with a lower Mendelian ratio and showed failure to thrive, which was particularly evident after weaning, suggesting that the metabolic disturbance exacerbates with the switch in diet from breast milk to regular chow. We measured body mass at 6, 7, 8, and 9 weeks and confirmed a previously described growth arrest (Vidali et al. 2021) resulting in significantly lower body mass of approximately 75% (or 4–5 g less) at 6 weeks and approximately 65% (or 7–9 g less) at 9 weeks of age compared to wildtype littermate (C57BL/6 N) control mice (Table 1). The growth arrest was accompanied by significantly elevated blood glucose levels in *Uqcrh*-KO with > 25 mmol/L at 6 weeks of age, which further increased to > 29 mmol/L at 9 weeks of age compared to 3.4–9.7 mmol/L measured throughout in wildtype littermate controls (Table 1).

**Table 1:** Body mass and tail blood glucose levels of wildtype littermate controls (C57BL/6 N) and *Uqcrh*-KO mutant mice by sex and time points as indicated

	Female		Male	
	C57BL/6N	*Uqcrh*-KO	C57BL/6N	*Uqcrh*-KO
	*n* = 15	*n* = 15	*n* = 15	*n* = 15
	median	median	median	median
	[25%, 75%]	[25%, 75%]	[25%, 75%]	[25%, 75%]
Week 6				
Glucose [mmol/l]	6.99	27.75	8.82	25.25
	[6.86, 7.5]	[22.18, 31.36]	[8.29, 9.1]	[23.81, 32.99]
Body mass [g]	17.10	13.00	19.30	14.30
	[15.8, 17.7]	[11.8, 13.4]	[18.2, 20.6]	[14.2, 14.8]
Week 7				
Glucose [mmol/l]	7.60	29.36	7.60	26.36
	[7.24, 8]	[26.23, 32.88]	[6.77, 8.04]	[25.61, 28.52]
Body mass [g]	18.10	12.90	21.60	14.30
	[17.1, 19.1]	[12.2, 13.7]	[19.2, 22.4]	[13.8, 14.8]
Week 8				
Glucose [mmol/l]	7.27	29.08	8.21	29.30
	[7.02, 7.74]	[27.39, 30.55]	[7.71, 8.63]	[27.59, 31.19]
Body mass [g]	19.40	12.90	22.60	14.70
	[18, 19.9]	[12.3, 13.6]	[20.8, 23.8]	[14.3, 15.2]
Week 9				
Glucose [mmol/l]	6.99	29.36	8.05	30.75
	[6.69, 7.66]	[27.2, 31.25]	[7.68, 8.55]	[28.3, 33.49]
Body mass [g]	20.00	13.10	24.30	15.10
	[18.8, 21]	[12.4, 13.8]	[22.3, 25]	[14.8, 15.6]

Data shown as median with confidence intervals (25/75%) in brackets

### *Uqcrh*-KO hearts show no gross histopathologic alterations

**Fig. 1:**
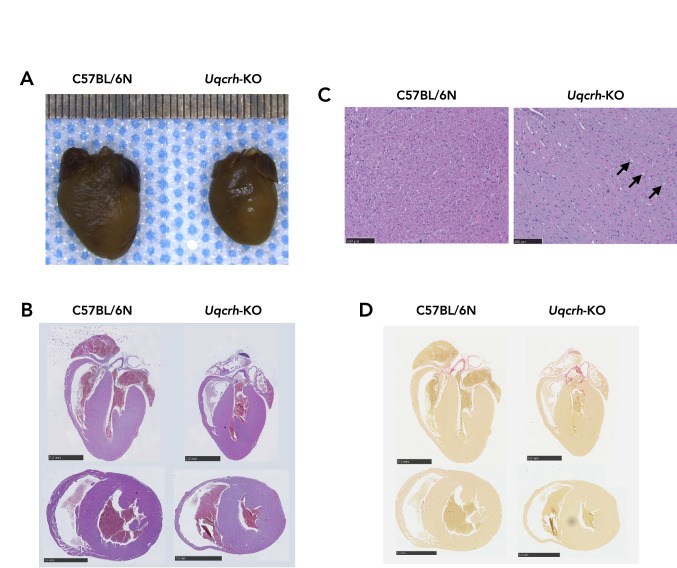
Histopathological analysis of hearts from 10-week-old *Uqcrh*-KO mutant mice and wildtype littermate controls. **A** Representative formalin-fixed hearts from mice with genotype as indicated. One mark on the ruler above the hearts corresponds to 0.5 mm. **B** Representative heart slices, as shown in **A**, sectioned longitudinally and transversely, stained with hematoxylin and eosin (H&E). Black bars represent 2.5 mm. **C** H&E stain of representative myocardium, as shown in **B** at higher magnification. Note the presence of vacuoles (black arrows) particularly in heart tissue of *Uqcrh*-KO mice. Black bars represent 100 µm. **D** Representative heart slices, sectioned longitudinally and transversely, stained with Sirius Red to stain for collagen deposition. Black bars represent 2.5 mm

We next examined the extent to which the heart, as a primarily oxidative organ, was macro- and microscopically affected by *Uqcrh* gene deletion and CIII impairment. At 10 weeks of age, hearts from *Uqcrh*-KO mice were consistently smaller compared with hearts from wildtype littermate controls (Fig. 1A–D). At higher magnification, H&E-stained heart sections (Fig. 1B, C) showed no gross morphological abnormalities in the size of the ventricles, heart valves, and large vessels at the base of the heart, except for the appearance of some vacuoles in the cardiomyocytes of *Uqcrh*-KO mutant mice. The appearance of such vacuoles has previously been described as a histopathological sign for a wide range of pathological conditions, ranging from toxic stress and troponin loss to mitochondrial damage due to impaired lipid oxidation (Dunnick et al. 2004a, b; Jokinen et al. 2004). Interestingly, Sirius Red staining presented no excess deposition of interstitial collagen fibers in *Uqcrh*-KO hearts (Fig. 1D).

### Similar absolute LV morphology in *Uqcrh*-KO and wildtype littermate control mice

**Fig. 2:**
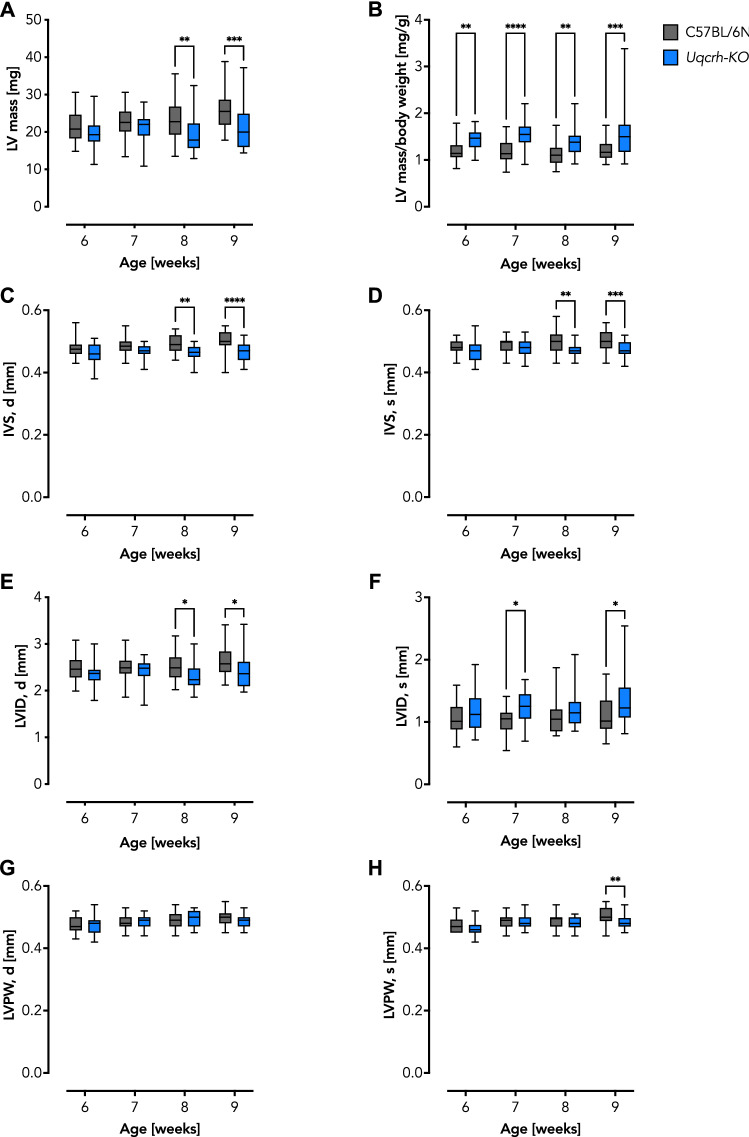
Left ventricular geometry assessed by echocardiographic reveals only subtle differences at selected time points despite the marked decrease in overall organ size in *Uqcrh*-KO mice. **A** Left ventricular (LV) mass as assessed by echocardiography for wildtype littermate controls and *Uqcrh*-KO mice as indicated. **B** Calculated heart mass normalized to body mass. Note the developmental stalling and markedly decreased body mass in *Uqcrh*-KO mice upon weaning (Table 1). **C** Diastolic interventricular septum (IVS, d) thickness. **D** Systolic interventricular septum (IVS, s) thickness. **E** Diastolic left ventricular inner dimension (LVID, d). **F** Systolic left ventricular inner dimension (LVID, s). **G** Diastolic left ventricular posterior wall thickness (LVPW, d). **H** Systolic left ventricular posterior wall thickness (LVPW, s). Data (*n* ≥ 21) are shown as box and whisker plots indicating the position of the minimum, lower quartile, median, upper quartile and maximum. *P < 0.05, **P < 0.01, ***P < 0.001, ****P < 0.0001 analyzed by 2way ANOVA with *post-hoc* Šídák's multiple comparisons test using Prism 9 (GraphPad Software)

We performed transthoracic echocardiography (TTE) to examine cardiac geometry in vivo (Suppl. Tables S1–4) and found a similar left ventricular (LV) mass at 6 and 7 weeks of age. At 8 and 9 weeks of age, however, a significant, albeit marginal, decrease in LV mass became evident in *Uqcrh*-KO mutants compared with wildtype littermate controls (Fig. 2A). The absolute LV mass suggests that the difference was mainly due to the aforementioned postnatal developmental arrest in the *Uqcrh*-KO mice. The LV mass expressed relative to body mass did not change with age within either group (Fig. 2B). However, the relative LV mass was significantly higher in the *Uqcrh*-KO mutant mice compared to wildtype littermate controls (Fig. 2B). Although the absolute difference was subtle, this may indicate that *Uqcrh*-KO mutant mice develop a mild form of cardiac enlargement in relation to their body mass. In addition, we found a decreased thickness of the interventricular septum in *Uqcrh*-KO mutant mice in diastole and systole at 8 and 9 weeks (Fig. 2C, D), whereas other parameters such as LV internal dimension (LVID) and LV posterior wall thickness (LVPW) revealed incidental differences without a clear tendency (Fig. 2E–H). Overall, the absolute mass showed only minimal morphological differences with a tendency to decrease in *Uqcrh*-KO mutant mice, but this tendency was reversed when related to body mass.

### *Uqcrh*-KO mutant hearts develop a contractile malfunction

**Fig. 3:**
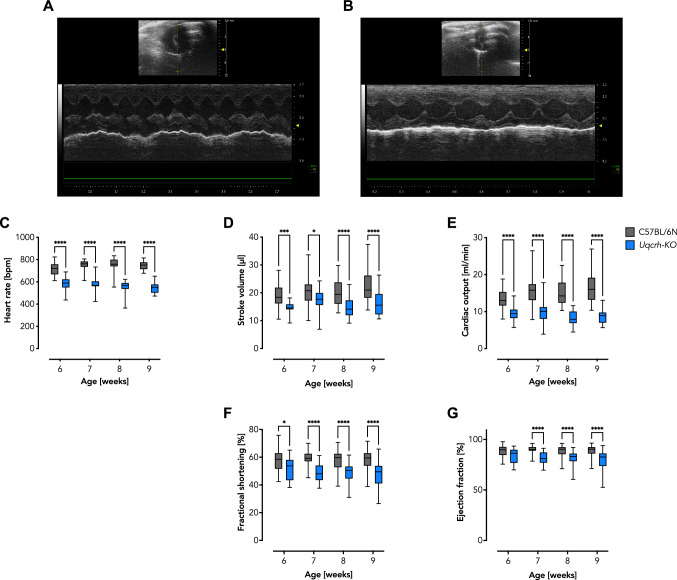
Left ventricular contractile function assessed by m-mode short axis transthoracic echocardiographic reveals cardiac contractile malfunction in *Uqcrh*-KO. **A** Representative echocardiographic trace of a female wildtype littermate control mouse at 9 weeks of age. **B** Representative echocardiographic trace of a female *Uqcrh*-KO mouse at 9 weeks of age. **C** Heart rate as monitored during echocardiography. **D** Calculated stroke volume. **E** Calculated cardiac output. **F** Calculated fractional shortening. **G** Calculated ejection fraction. Data (*n* ≥ 21) are shown as box and whisker plots indicating the position of the minimum, lower quartile, median, upper quartile and maximum. *P < 0.05, ***P < 0.001, ****P < 0.0001 analyzed by 2way ANOVA with post-hoc Šídák's multiple comparisons test using Prism 9 (GraphPad Software)

Based on TTE recordings, we investigated cardiac functions such as heart rate and contractility and found that heart rate was significantly decreased in the *Uqcrh*-KO mutant mice compared to wildtype littermate controls at all timepoints (Fig. 3A–C). Moreover, several parameters were similarly decreased with a clear tendency to deteriorate over time, namely, stroke volume (SV, Fig. 3D), cardiac output (CO, Fig. 3E), fractional shortening (FS, Fig. 3F), and ejection fraction (EF, Fig. 3G). In summary, these TTE data suggest a development of contractile dysfunction in *Uqcrh*-KO mutant mice.

### *Uqcrh*-KO cardiac mitochondria exhibit decreased respiratory capacity

**Fig. 4:**
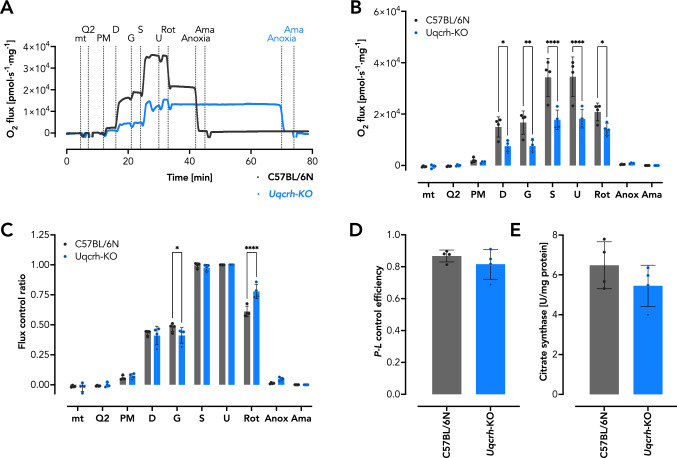
Isolated *Uqcrh*-KO heart mitochondria show decreased O_2_ consumption despite high coupling efficiency (measured simultaneously with the redox state of the ETS-reactive Q). **A** Representative traces of respiring isolated cardiac mitochondria from wildtype littermate controls and *Uqcrh*-KO mice in the presence of substrates and inhibitors as indicated. *mt* mitochondria, *PM* NADH-linked substrates pyruvate and malate, *D* adenosine diphosphate (ADP), *G* NADH-linked substrate glutamate, *S* succinate, *U* uncoupler carbonyl cyanide m‐chlorophenylhydrazone (CCCP), *Rot* Complex I inhibitor rotenone, *Anoxia* depletion of O_2_, *Ama* Complex III inhibitor antimycin A. **B** O_2_ flux per mass (*n* = 4), i.e., the negative time derivative of the O_2_ concentration automatically calculated by DatLab 7.4 software (Oroboros Instruments, Innsbruck, Austria), corrected for instrumental background, and normalized for mitochondrial protein concentration. **C** Flux control ratio (*FCR*) (*n* = 4), i.e., ratios of O_2_ flux in different respiratory states normalized to the maximum flux in a common reference state here upon uncoupling (U) where 1 is maximal respiratory rate (100%) (Gnaiger 2020; Gnaiger and MitoEAGLE-Task-Group 2020). *FCR* serves as a control benchmark for coupling and substrate control, independent of mitochondrial content and purification. **D** *P-L* control efficiency (*n* = 4), i.e., OXPHOS capacity corrected for LEAK respiration (net *P-L* OXPHOS capacity) normalized to total OXPHOS capacity *P*, used as a mitochondrial quality control, where 1 is fully coupled and 0 is zero coupled with zero phosphorylation capacity (Gnaiger 2020; Gnaiger and MitoEAGLE-Task-Group 2020). **E** Activity of the mitochondrial marker enzyme citrate synthase in isolated heart mitochondria (*n* = 4). Data are shown as mean ± SD, *P < 0.05, **P < 0.01, ****P < 0.0001 analyzed by 2way ANOVA with *post-hoc* Šídák's multiple comparisons test using Prism 9 (GraphPad Software)

To test the function of the ETS, we isolated cardiac mitochondria from the LV and used the NextGen-O2k that allows simultaneous assessment of mitochondrial respiration with either the ETS-reactive Q redox state or mitochondrial ROS (H_2_O_2_) production in two chambers, i.e., we measured a combination of the three parameters in two independent experiments. The respiration rate decreased significantly in *Uqcrh*-KO mutant mitochondria (Fig. 4; Suppl. Fig. S1). Using combined measurements of respiratory capacity and Q redox state, we found no difference between NADH-linked pyruvate (P) and malate (M)-driven LEAK respiration, i.e., the non-phosphorylating resting state that compensates for proton leak, proton slip, cation exchange, and electron leak was similar in both genotypes (Gnaiger 2020; Gnaiger and MitoEAGLE-Task-Group 2020) (Fig. 4A, B). Conversely, OXPHOS respiration was lower in *Uqcrh*-KO mutant mitochondria after addition of adenosine diphosphate (D) driven by P and M alone plus after addition of glutamate (G) (N-linked pathway) and succinate (S) (NS-linked pathway) (Fig. 4A, B). The maximum electron transfer (ET) capacity after titration of the uncoupler carbonyl cyanide m-chlorophenylhydrazone (U) and the S-linked ET capacity after the CI inhibitor rotenone (Rot) was added were equally decreased in the two groups (Fig. 4A, B). In this approach, respiration was terminated by reaching anoxia, which took longer in mitochondria of *Uqcrh*-KO mutants because of the lower respiration. Both reaching anoxia and adding antimycin A (Ama) are important control measures to assess the Q redox state (Komlódi et al. 2021a). To test whether the observed differences in respiratory capacity were due to differences in quality between mitochondrial preparations or to experimental design, we calculated markers of quality control, i.e., flux control ratio (*FCR*) and *P-L* control efficiency (Fig. 4C, D). *FCR* is generally used as a control measure for coupling and substrate control, independent of mitochondrial content and purification plus assay conditions. It is the ratio of oxygen flux in different respiration states normalized to the maximum flux in a common reference state, here at U, where the *FCR* equals the maximum of 1 (100%) (Gnaiger 2020; Gnaiger and MitoEAGLE-Task-Group 2020). In particular, the *FCR* values showed a subtle but significant increase of the relative S-linked ET capacity in the mitochondria of *Uqcrh*-KO mutant mice (Fig. 4C), a result consistent with the concept of improved substrate availability or electron shuttling due to altered CIII assembly. In addition, we calculated *P-L* control efficiency, which is the ratio of *P-L* net OXPHOS capacity (OXPHOS capacity minus LEAK respiration) to total OXPHOS capacity, another measure of mitochondrial quality control, with 1 representing fully coupled OXPHOS capacity and 0 representing no respiratory phosphorylation capacity (Gnaiger 2020). The *P-L* control efficiency revealed no difference between *Uqcrh*-KO mutants and wildtype littermate controls (Fig. 4D). Finally, we measured the activity of the mitochondrial marker enzyme citrate synthase, which showed no differences (Fig. 4E). These results indicate that the overall quality of mitochondrial preparations was comparable. Moreover, similar results were observed in the parallel assessment of respiratory capacity and H_2_O_2_ production (Suppl. Fig. S1).

### Respiring *Uqcrh*-KO mitochondria show a reduced Q redox state but no excessive ROS production

**Fig. 5:**
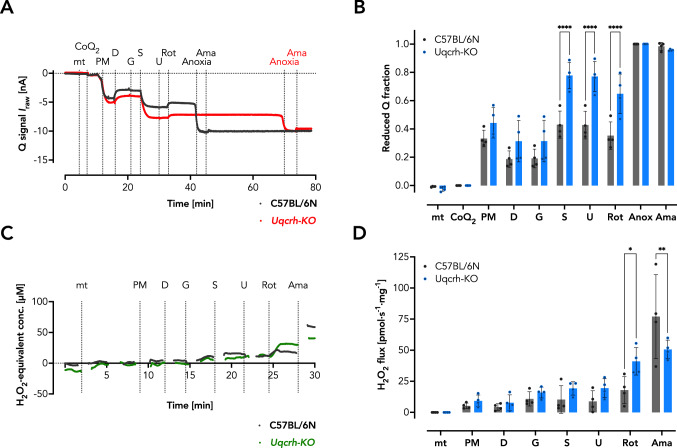
Isolated *Uqcrh*-KO heart mitochondria show a greater degree of ETS-reactive Q reduction but no increase in H_2_O_2_ production during O_2_ consumption in the absence of inhibitors. **A** Representative traces of the Q redox state in respiring isolated cardiac mitochondria from wildtype littermate controls and *Uqcrh*-KO mice in the presence of substrates and inhibitors as indicated. *mt* mitochondria, *CoQ*_*2*_ 2,3-dimethoxy-5-methyl-6-geranyl-1,4-benzoquinone, *PM* NADH-linked substrates pyruvate and malate, *D* adenosine diphosphate (ADP), *G* NADH-linked substrate glutamate, *S* succinate, *U* uncoupler carbonyl cyanide m‐chlorophenylhydrazone (CCCP), *Rot* Complex I inhibitor rotenone, *Anoxia* depletion of O_2_, *Ama* Complex III inhibitor antimycin A. **B** Quantification of the Q redox state. **C** Representative traces of H_2_O_2_ production in respiring isolated cardiac mitochondria from wildtype littermate controls and *Uqcrh*-KO mice in the presence of substrates and inhibitors as indicated. **D** Quantification of H_2_O_2_ production. Data are shown as mean ± SD, *P < 0.05, **P < 0.01, ****P < 0.0001 analyzed by 2way ANOVA with *post-hoc* Šídák's multiple comparisons test using Prism 9 (GraphPad Software)

Finally, we tested whether the decrease in respiratory capacity affects the Q redox state and possibly causes an excessive production of ROS. In particular, the study of the Q redox state provides interesting information because it reflects the sum of electron influx into and efflux from Q (i.e., the overall electron flux through the ETS). We assumed that a defect in CIII impairs electron efflux and thus causes a reduced Q state in *Uqcrh*-KO mutant mitochondria. Indeed, we found the Q was more reduced, especially under conditions of S-linked respiration (Fig. 5A, B). This is consistent with previous data and would suggest that more ROS is produced upon succinate oxidation in the LEAK state in mitochondria of *Uqcrh*-KO mutants (Robb et al. 2018; Szibor et al. 2020a). During regular respiration in the absence of inhibitors, ROS production was similar in mitochondria of *Uqcrh*-KO mutant mice and wildtype littermate controls. After addition of the CI inhibitor Rot, *Uqcrh*-KO mitochondria produced more ROS than control mitochondria (Fig. 5C, D), whereas subsequent addition of the CIII inhibitor Ama resulted in excessive ROS production in mitochondria of wildtype littermate controls, with no additional effect in *Uqcrh*-KO (Fig. 5C, D).

## Discussion

The heart is a high-energy demanding organ that relies almost exclusively on OXPHOS for ATP production, a process facilitated by the ETS. Impairment of the ETS, *e.g.*, in the course of mitochondrial diseases, forces the heart to switch metabolism to less efficient glycolysis and at the same time to initiate remodeling processes that may collectively lead to a malfunction of cardiac contraction (Verdejo et al. 2012; Jia et al. 2016; Gorman et al. 2016; Zhou and Tian 2018; Bertero and Maack 2018). A prime example of this close relationship between ETS function and contractile adaptation is the development of lethal cardiomyopathy in CIII-deficient *Bcs1l*^*p.S78G*^ mice, which can be rescued when a compromised CIII is bypassed by alternative oxidase (AOX) (Rajendran et al. 2019). Here, we set out to investigate the effects of another rare CIII defect, a partial deletion of the *Uqcrh* gene, on cardiac morphology, contractile function, and mitochondrial bioenergetics. To account for systemic metabolic signaling as present in patients, we chose a global *Uqcrh* gene ablation, the previously described *Uqcrh*-KO mutant mouse (Vidali et al. 2021). *Uqcrh*-KO mutant mice presented with failure to thrive predominantly after weaning concomitant with extensively elevated blood glucose levels (Table 1). These observations are indicative for a major metabolic disorder development as previously reported (Vidali et al. 2021). Further, gross heart size was decreased in *Uqcrh*-KO mutant mice, which is not surprising given the overall phenotype (Fig. 1). TTE-mediated assessment of in vivo heart geometry confirmed decreased LV mass (Fig. 2A). Further, we observed relatively similar values for wall thickness and LV internal diameter, which nevertheless reached statistically significant differences at 8 and 9 weeks of age in some cases (Fig. 2C–H). It is likely that the low LV dimensions relate to the growth arrest and severely decreased body size and mass observed in *Uqcrh*-KO mice. This assumption is supported by the fact that the body mass plateaued in *Uqcrh*-KO mutant mice, whereas wildtype littermate control mice showed an expected growth curve. These results prompted us to relate heart mass calculated from TTE assessment to body mass, which revealed an increased ratio in the *Uqcrh*-KO mutant mice, a finding suggesting the development of cardiac enlargement (Fig. 2B). Of note, *Uqcrh*-KO mutant mice were not only smaller than wildtype littermate controls (Table 1) but also exhibited a different body composition (Vidali et al. 2021), i.e., they had higher fat mass and lower lean mass relative to the overall lower body mass. Interestingly, the calculated cardiac enlargement (Fig. 2B) was not accompanied by excess of collagen deposition (Fig. 1D). Instead, histopathological analysis revealed no gross morphological abnormalities (Fig. 1), except for the observation of mild vacuolization in the myocardium (Fig. 1C), a finding previously described as a sign of mitochondrial dysfunction (Dunnick et al. 2004a, b; Jokinen et al. 2004). TTE also assessed real-time cardiac function. Interestingly, we found a significantly lower heart rate in the *Uqcrh*-KO mutant mice compared with wildtype littermate controls (Fig. 3A–C), which, together with the decreased stroke volume (Fig. 3D), explained the calculated decrease in cardiac output (Fig. 3E). Clinically important landmarks for proper heart function such as fractional shortening and ejection fraction were also decreased in *Uqcrh*-KO mutants compared to wildtype littermate controls (Fig. 3F, G). Overall, our data suggest the presence of profound contractile dysfunction in a structurally regular heart at almost all time points but with a clear tendency of functional deterioration over time.

We reasoned that an impaired respiratory capacity due to the CIII defect might be the underlying cause of the observed contractile dysfunction. Using the newly developed NextGen-O2k (Oroboros Instruments, Innsbruck, Austria; (Komlódi et al. 2021a)) that allows high-resolution respirometry to be measured simultaneously with Q redox state or mitochondrial ROS production, we detected a significant decrease in mitochondrial respiratory capacity in cardiac mitochondria isolated from *Uqcrh*-KO mutant mice compared with controls. Likewise, ETS-reactive Q was more reduced in the mutant mice, suggesting an impairment of electron flux through the ETS, as expected. Interestingly, measurement of the *FCR* in different approaches showed that relative S-linked respiration in the presence of Rot was significantly increased in mutant mitochondria only. This small albeit significant difference might hint that electrons entering the ETS through the S-linked pathway can more easily reach CIII. Notably, the functional CIII is organized as a dimer (CIII_2_) and we have previously shown that *Uqcrh*-KO mutant mice lack CIII_2_ (Vidali et al. 2021). Our data possibly support the concept of different CoQ pools acting independently within the ETS, or a controlled oxidation of reduced Q dependent on supercomplex formation, here unleashing S-linked respiration. Equally surprising was the finding that in the absence of respiratory inhibitors, *Uqcrh*-KO mitochondria did not produce more ROS than wildtype littermate controls (Fig. 5C) despite the observed decrease in respiratory capacity (Fig. 4A, B; Suppl. Fig. S1A, B) and increase in the reduced Q fraction (Fig. 5A, B). This may explain the absence of cell damage or remodeling such as increased collagen deposition revealed in the histopathologic analysis of the hearts (Fig. 1). Yet, the decrease of respiratory capacity may be the underlying reason for the appearance of tissue vacuolization (Fig. 1C).

Another unexpected finding was that addition of Rot specifically increased mitochondrial ROS production in *Uqcrh*-KO mutant mitochondria, while the CIII inhibitor Ama increased mitochondrial ROS production in wildtype littermate controls without exhibiting an additive effect in mutants (Fig. 5C, D). We reasoned that in *Uqcrh*-KO Rot induces ROS at CIII in a forward flux, i.e., by electrons fueled by the S-linked pathway. The observed effect is unlikely due to a lack of Ama function since it showed efficient inhibition of oxygen consumption (Suppl. Fig. S1B, D). We thus conclude that the observed ROS may originate from two distinct sources, i.e., CIII and CIII_2_ whereas CIII_2_, only present in wildtype littermate controls, may be more efficient in producing ROS.

The lack of ROS production in the absence of respiratory inhibitors may be an interesting observation in another context. Elevated blood glucose levels, as observed in our *Uqcrh*-KO mutant mice or in patients with *Diabetes mellitus*, have previously been associated with excessive ROS production, and mitochondria were proposed as the major source. This does not appear to be the case when CIII is not dimerized, suggesting that ROS sources other than mitochondria are more important or that toxic effects outside the mitochondrial compartment are essential. Nevertheless, the presence of highly elevated glucose levels in combination with failure to thrive and impaired ETS in mitochondria may therefore make *Uqcrh*-KO mutant mice a valuable in vivo model for further in-depth investigation of mitochondrial diabetic cardiomyopathy and other multisystemic pathologies associated with hyperglycemia, such as sepsis.

In summary, we demonstrate that global deletion of the murine *Uqcrh* gene induces hyperglycemia and postnatal developmental arrest that becomes particularly evident after weaning. The absolute heart size is, like the body mass, decreased but gross heart geometry and morphology appear unaffected. Relating heart mass to body mass indicates the development of organ enlargement in *Uqcrh*-KO mutant mice in the absence of adverse collagen deposition. Assessment of cardiac contractile and mitochondrial functions revealed a marked decrease in mitochondrial respiratory capacity and a more reduced Q redox state in *Uqcrh*-KO mutant mice indicating that electron flux through the ETS is indeed impaired. In the absence of respiratory inhibitors, this ETS impairment, however, is not paralleled by excessive production of mitochondrial ROS suggesting that the observed cardiac contractile malfunction is primarily caused by ATP depletion or redox imbalance but not ROS-mediated cell damage or death.

## Methods

### Generation of the *Uqcrh*-KO mouse strain and phenotyping

The *Uqcrh* knockout mouse strain (*Uqcrh*-KO, *C57BL/6NCrl-Uqcrh*^*tm1b(EUCOMM)Wtsi*^/Ieg) was generated by the International Mouse Phenotyping Consortium (IMPC) on a C57BL/6N (wildtype) background by allele conversion of the *C57BL/6NCrl-Uqcrh*^*tm1a(EUCOMM)Wtsi*^/Ieg mouse line derived from the EUCOMM ES cell clone EPD0378_3_C07 as previously described (Ryder et al. 2014; Vidali et al. 2021). Further details on genomic manipulations are available online at the IMPC portal (https://www.mousephenotype.org/data/alleles/MGI:1913826/tm1b(EUCOMM)Wtsi). Briefly, in *Uqcrh*-KO mice, exons two and three of the *Uqcrh* gene are deleted by integrating a LacZ cassette. This LacZ is transcriptionally fused to exon one and thus expressed under the control of the exogenous *Uqcrh* promoter (Friedel et al. 2010). This results in a true gene ablation, as skipping the LacZ cassette does not generate a functional UQCRH protein. Generated mice were genotyped to verify the two-exon deletion following genotyping protocols freely available on the Infrafrontier website (https://www.infrafrontier.eu/sites/infrafrontier.eu/files/upload/public/pdf/genotype_protocols/EM10141_geno.pdf). Heterozygous *Uqcrh*-KO mice were then crossbred to generate homozygous mutants. General phenotypic analysis was done as detailed below and previously described (Fuchs et al. 2009, 2011, 2018).

### Housing conditions and animal welfare

All mice were maintained in IVC cages with water and standard chow ad libitum according to the directive 2010/63/EU and German laws. Homozygous *Uqcrh*-KO mice and their wildtype littermate (C57BL/6N) controls were fed with moist food in addition to the regular chow. All procedures were performed following standard protocols (www.mouseclinic.de) upon approval by responsible authorities, i.e., the District Government of Upper Bavaria and the Thüringer Landesamt für Verbraucherschutz (TLV, UKJ-18-033).

### Transthoracic echocardiography

Transthoracic echocardiography (TTE) has been performed essentially as described in detail elsewhere (Moreth et al. 2014). Briefly, TTE was done weekly between weeks 6 and 9 of age in conscious mice using a Vevo3100 Imaging System (VisualSonics, Toronto, Canada) with a 30 MHz probe. A total of 60 animals were examined, i.e., 15 males and females of *Uqcrh*-KO mutants and 15 males and females of wildtype littermate controls. To minimize circadian influences, all TTE examinations were done in the morning between 8 and 11 am. Mice were allowed to familiarize themselves with the experimental area for at least 30 min before measurements. All experiments were performed in a conditioned, quiet room to avoid external stimuli that might affect the physiology of the mice. Notably, no anesthesia was used to exclude any impairment of cardiac function (Roth et al. 2002). All echocardiograms were recorded and analyzed by the same person, blinded to the mouse genotype.

### Body mass and blood glucose levels

Body mass was determined immediately before TTE. Tail blood glucose levels were determined immediately after TTE using Nova Biomedical StatStrip Xpress Glucose Strips and the corresponding Nova Biomedical handheld glucose meter. To collect blood drops, the tail vein was punctured with a sterile cannula or lancet, and hemostasis was achieved by compression.

### Histopathology

For histopathological analyses of the heart, 12 *Uqcrh*-KO mice (6 males and 6 females) and eight wildtype littermate controls (4 males and 4 females) at 10 weeks of age were included. Hearts were fixed in formalin and embedded in paraffin for further examination. Longitudinal and transverse sections 3-µm thick were made to visualize the ventricles, valves, papillary muscles, and major vessels at the base of the heart. These landmarks were used to ensure comparable anatomic regions. Because of regulatory restrictions, final blood withdrawal and determination of cardiac weight and tibia length were not possible. Therefore, these data could not be used to validate data obtained by TTE and/or for normalization. Lack of final exsanguination is why blood remnants are seen in the cardiac images. Heart sections were stained with hematoxylin and eosin (H&E) for general morphology evaluation and with Sirius red for collagen deposition, as described previously (Fuchs et al. 2009, 2011, 2018). Sections were scanned using a digital slide scanner (NanoZoomer, Hamamatsu, Japan). Histopathologic evaluation was performed by two trained pathologists.

### Isolation of heart mitochondria

Heart mitochondria from 12-week-old mice were isolated as previously described (Szibor et al. 2020a, 2022). Briefly, hearts were rapidly excised, right ventricle and atria removed and transferred to ice-cold MMSE-A buffer (225 mM d-mannitol, 20 mM MOPS, 75 mM sucrose, 1 mM ethylene glycol-bis(2-aminoethylether)-*N*,*N*,*N*′,*N*′-tetraacetic acid (EGTA), 0.5 mM dl-dithiothreitol (DTT), pH 7.4). All further steps were performed on ice. Heart tissues were minced using scissors and manually pottered in a glass-on-Teflon homogenizer until homogenous in MMSE-B buffer (MMSE-A buffer plus 0.05% nagarse). Nagarse activity was stopped by 1:30 dilution of the homogenate in MMSE-A buffer. The homogenate was centrifuged at 2 000×*g* for 4 min at 4 °C and the supernatant passed through cheesecloth. The flow-through was centrifuged at 12 000×*g* for 10 min at 4 °C. The fluffy layer of the pellet was removed and discarded. The mitochondrial dense layer of the pellet was resuspended for further testing in ice-cold KME buffer (100 mM potassium chloride, 0.5 mM EGTA, pH 8.5). Subsequently, mitochondrial protein concentration was determined by the Bradford method using bovine serum albumin as a standard (Protein Assay Dye Reagent Concentrate, Bio-Rad, #5000006; protein standard, Sigma, P0834).

### High-resolution respirometry

O_2_ concentration, O_2_ flux, H_2_O_2_ flux, and the redox state of ETS-reactive coenzyme Q (Q) were measured using the NextGen-O2k (Oroboros Instruments, Innsbruck, Austria; (Komlódi et al. 2021a)). The experiments were performed in a 2-mL chamber under constant stirring (750 rpm) at 37 °C in the mitochondrial respiration medium MiR05 (0.5 mM EGTA, 3 mM MgCl_2_, 60 mM lactobionic acid, 20 mM taurine, 10 mM KH_2_PO_4_, 20 mM HEPES, 110 mM d-sucrose, 1 g/L fatty acid free bovine serum albumin; pH 7.1; Oroboros Instruments). The O_2_ concentration was monitored with polarographic O_2_ sensors (POS), and the O_2_ flux was calculated as the negative time derivative of the O_2_ concentration real-time by the DatLab 7.4 software (Oroboros Instruments). Air calibration including a stirrer test and an instrumental O_2_ background test including zero calibration of the POS were performed routinely as part of the instrumental quality control (Gnaiger 2001, 2008). The observed minimal instrumental O_2_ background flux is caused by O_2_ consumption of the POS and due to O_2_ diffusion into and out of the O2k-chamber. O_2_ flux was corrected for (***1***) instrumental O_2_ background flux, (***2***) dilution of the sample by titrations, (***3***) residual O_2_ consumption, *Rox*, and normalized for mitochondrial protein concentration (pmol s^−1^ mg^−1^).

### ETS-reactive Q redox state measurements

The Q redox state was monitored with the Q-Module of the NextGen-O2k as previously described (Szibor et al. 2022). Briefly, a three-electrode system and a CoQ mimetic, coenzyme Q_2_ (CoQ_2_; 1 µM) are required to follow the redox changes of mitochondrial Q (Komlódi et al. 2021a). The three-electrode system consists of a working electrode (glassy carbon), a counter electrode (Pt; platinum), and a reference electrode (RE; Ag/AgCl). CoQ_2_ reacts both with the glassy carbon working electrode and with the mitochondrial Complexes (Szibor et al. 2022). At equilibrium, the ratio of oxidized and reduced external CoQ_2_ is the same as the ratio of oxidized and reduced endogenous mitochondrial ETS-reactive Q. In this case, the CoQ_2_ redox state reflects the redox state of mitochondrial Q (Moore et al. 1988; Bergen et al. 1994). The fully oxidized and fully reduced CoQ_2_ were measured in every experiment to calculate the reduced Q fraction as previously described (Komlódi et al. 2021a). The fully oxidized CoQ_2_ was monitored in the presence of isolated mitochondria and CoQ_2_ (1 µM) but in the absence of respiratory fuel substrates and ADP. The fully reduced CoQ_2_ was recorded under anoxia. Cyclic voltammetry was applied to determine the oxidation peak potential of CoQ_2_ (Komlódi et al. 2021a). The glassy carbon was poised at the oxidation peak potential to oxidize the reduced CoQ_2_. The current was proportional to the concentration of reduced CoQ_2_, thus, the current increased as the concentration of reduced CoQ_2_ increased.

### Hydrogen peroxide (H_2_O_2_) flux

Hydrogen peroxide (H_2_O_2_) flux was measured with the Amplex™ UltraRed assay simultaneously with high-resolution respirometry using Smart-Fluo Sensors Green (excitation at 525 nm) in MiR05 (Krumschnabel et al. 2015; Komlódi et al. 2018, 2021b). Amplex UltraRed (AmR; 10 µM) reacts with H_2_O_2_ in a reaction catalyzed by horseradish peroxidase (HRP; 1 U/mL) forming the fluorescent Amplex UltroxRed. Superoxide dismutase (SOD; 5 U/mL) was added to avoid undesirable side effect of the AmR assay with NADH and glutathione (Votyakova and Reynolds 2004) and to transform all superoxide produced outside the mitochondrial matrix into H_2_O_2_. Diethylenetriamine-*N*,*N*,*N*′,*N*′′,*N*′′-pentaacetic acid (DTPA; 15 µM) was injected into the O2k-chamber before sample addition to chelate iron and therefore decrease the fluorescence background flux of the AmR assay (Komlódi et al. 2018). The fluorescence signal was calibrated with multiple titrations of 0.1 µM H_2_O_2_ to monitor the sensitivity of the AmR assay toward H_2_O_2_ (Komlódi et al. 2018). The fluorescence slope (*I*_*Amp*_) was calculated as the time derivative of the fluorescence signal by DatLab 7.4. *I*_*Amp*_ was corrected for (***1***) sensitivity (µA µM^−1^), (***2***) dilution of the sample by titrations, and (***3***) normalized for mitochondrial protein concentration (pmol s^−1^ mg^−1^) (Komlódi et al. 2021b).

### Experimental procedure

Isolated mitochondria were injected into the O2k-chamber directly after the isolation procedure with a 50-µL Hamilton syringe (0.005 mg/mL). Pyruvate (P; 5 mM) and malate (M; 2 mM) were added as respiratory substrates to monitor NADH-linked (N) LEAK respiration, the non-phosphorylating resting state compensating for proton leak, proton slip, cation cycling, and electron leak (Gnaiger 2020; Gnaiger and MitoEAGLE-Task-Group 2020). It was followed by the addition of kinetically saturating concentration of adenosine diphosphate (ADP, D; 2 mM) to measure OXPHOS capacity. Afterward, glutamate (G; 10 mM) was injected to measure PGM-linked OXPHOS capacity and succinate (S; 10 mM) to monitor NS-linked OXPHOS capacity. The uncoupler, carbonyl cyanide m-chlorophenyl hydrazone (CCCP; 0.5 µM) was titrated stepwise to record maximal electron transfer (ET) capacity which did not further increase the O_2_ flux and thus indicated zero ET-excess capacity. The CI inhibitor rotenone (0.5 µM) was used to block N-linked respiration and to specifically monitor S-linked ET capacity. The CIII inhibitor antimycin A (Ama, 2.5 µM) was added to measure the residual O_2_ consumption (*Rox*) (Gnaiger 2020; Gnaiger and MitoEAGLE-Task-Group 2020). *P-L* control was calculated as the ratio of net OXPHOS capacity (corrected for LEAK respiration, i.e., *P-L*) and total OXPHOS capacity *P*, used as a mitochondrial quality control, where the maximum of 1 is obtained for fully coupled mitochondria and 0 indicates zero respiratory phosphorylation capacity. Flux control ratios (*FCR*) are the ratios of oxygen flux in different respiratory states normalized to the maximum flux in a common reference state, here in the NS-pathway in the OXPHOS- and ET-coupling state (Gnaiger 2020; Gnaiger and MitoEAGLE-Task-Group 2020). *FCR* serves as a control benchmark for coupling and substrate control, independent of mitochondrial content and purification.

### Citrate synthase activity

Citrate synthase activity was measured in freshly isolated mitochondria as previously described (Heyne et al. 2020). Briefly, mitochondria were diluted with KEA buffer (180 mM KCl; 10 mM EDTA; 0.5% BSA; pH 7.4). Citrate synthase activity was measured spectrophotometrically (*λ* = 412 nm) at 25 °C in a reaction medium containing 5,5′-dithio-bis(2-nitrobenzoic acid) (500 µM) in Tris–HCl (100 mM; pH 8.5) and acetyl-coenzyme A (125 µM). Maximal citrate synthase activity was measured by adding oxaloacetate (500 µM), and total citrate synthase activity was determined by incubating samples with Triton X-100 (5%).

### Statistical analyses

Statistical analyses were performed using Prism 9 (GraphPad Software). TTE data of *n* ≥ 21 are shown as box and whisker plots indicating the position of the minimum, lower quartile, median, upper quartile, and maximum. A *P* value < 0.05 analyzed by 2way ANOVA with *post-hoc* Šídák's multiple comparisons test was considered being statistically significant. High-resolution respirometry data of *n* = 4 are shown as mean with error bars representing standard deviations (SD). A *P* value < 0.05 analyzed by 2way ANOVA with *post-hoc* Šídák's multiple comparisons test was considered being statistically significant. The number of repeated experiments, individual *P* values, and statistical analyses applied are also given in each figure legend.

## Supplementary Information

Below is the link to the electronic supplementary material.

Supplementary file1 (DOCX 555 kb)

## Data Availability

The datasets used and/or analyzed during the current study, as well as the animal model used, are available from the corresponding author upon request.
