# Supplementary material for: Knockout of the Complex III subunit Uqcrh causes bioenergetic impairment and cardiac contractile dysfunction
Source: Mamm Genome. 2022 Dec 24;34(2):229–43. doi: 10.1007/s00335-022-09973-w (PMC10290608; doi:10.1007/s00335-022-09973-w)
Supplement: Supplementary file 1 — Supplementary file1 (DOCX 555 kb) [file 335_2022_9973_MOESM1_ESM.docx]

**Knockout of the Complex III subunit *Uqcrh* causes bioenergetic impairment and cardiac contractile dysfunction**

**Nadine Spielmann *et al.***

**Supplemental information**

Supplemental figure S1

Supplemental tables S1-S4

**Supplemental figure S1**. Isolated *Uqcrh*-KO heart mitochondria show decreased mitochondrial oxygen consumption despite regular coupling efficiency (measured simultaneously with hydrogen peroxide production using Amplex UltraRed). (**A**) Schematic of the adapted SUIT-008 protocol (https://wiki.oroboros.at/index.php/SUIT-008) (Lemieux et al. 2017) used throughout all experiments for high-resolution respirometry with a NextGen-O2k (Oroboros Instruments, Innsbruck, Austria). MiR, type of mitochondrial respiration; LEAK, non-phosphorylating resting state compensating for proton leak, proton slip, cation cycling and electron leak (Gnaiger 2020; Gnaiger and MitoEAGLE-Task-Group 2021); OXPHOS, oxidative phosphorylation of mitochondria in an ADP-activated state; ET, respiratory electron-transfer capacity; ROX, residual oxygen consumption due to oxidative side reactions; PM, NADH-linked substrates pyruvate and malate fueling the N-linked ET pathway; D, adenosine diphosphate (ADP); G, NADH-linked substrate glutamate; S, succinate fueling the S-linked ET pathway; U, uncoupling using carbonyl cyanide m‐chlorophenylhydrazone (CCCP); Rot, Complex I inhibitor rotenone; Ama, Complex III inhibitor antimycin A. (**B**) Representative traces of respiring isolated cardiac mitochondria from C57BL/6N and *Uqcrh*-KO mice in the presence of substrates and inhibitors as indicated. mt, mitochondria. (**C**) *P-L* control efficiency (*n* = 4), *i.e.*, OXPHOS capacity corrected for LEAK respiration (net P-L OXPHOS capacity) normalized to total OXPHOS capacity *P*, used as a mitochondrial quality control, where 1 is fully coupled and 0 is the zero coupled with zero phosphorylation capacity (Gnaiger 2020; Gnaiger and MitoEAGLE-Task-Group 2021). (**D**) Oxygen flux per mass (*n* = 4), *i.e.*, the negative time derivative of the oxygen concentration automatically calculated by DatLab 7 software (Oroboros Instruments, Innsbruck, Austria) normalized for mitochondrial protein concentration and corrected for instrumental background. (**E**) Flux control ratio (*FCR*) (*n* = 4), *i.e.*, ratios of oxygen flux in different respiratory states normalized to the maximum flux in a common reference state here upon uncoupling (U) where 1 is maximal respiratory rate (100%). *FCR* serves as a control benchmark for coupling and substrate control, independent of mitochondrial content and purification. Data are shown as mean ± SD, ***P* < 0.001, ****P* < 0.001 analyzed by 2way ANOVA with *post-hoc* Šídák's multiple comparisons test using Prism 9 (GraphPad Software).

**Supplemental table S1**. Transthoracic echocardiogram (TTE) data at 6 weeks of age. Data shown as median with confidence intervals (25/75%) in brackets. Abbreviations used: bpm, beats per minute; IVS, interventricular septum; LVPW, left ventricular posterior wall; LVID, left ventricular inner dimension; s, systolic; d, diastolic.

|  | Female | | Male | |
| --- | --- | --- | --- | --- |
|  | **C57BL/6N** | ***Uqcrh*-KO** | **C57BL/6N** | ***Uqcrh*-KO** |
|  | *n*=15 | *n*=15 | *n*=15 | *n*=15 |
|  | median | median | median | median |
|  | [25%, 75%] | [25%, 75%] | [25%, 75%] | [25%, 75%] |
| Heart rate [bpm] | 725.13 | 589.1 | 699.03 | 592.06 |
|  | [705.43, 760.34] | [555.66, 611.68] | [652.51, 754.78] | [551.27, 629.66] |
| Respiration rate [1/min] | 244.9 | 212.39 | 235.29 | 216.22 |
|  | [216.5, 269.8] | [185.34, 254.03] | [209.65, 259.83] | [194.34, 274.37] |
| IVS, s [mm] | 0.47 | 0.47 | 0.48 | 0.47 |
|  | [0.44, 0.48] | [0.44, 0.48] | [0.47, 0.5] | [0.44, 0.49] |
| IVS, d [mm] | 0.47 | 0.45 | 0.48 | 0.46 |
|  | [0.45, 0.48] | [0.44, 0.48] | [0.47, 0.49] | [0.44, 0.47] |
| LVPW, s [mm] | 0.47 | 0.45 | 0.47 | 0.47 |
|  | [0.46, 0.48] | [0.44, 0.46] | [0.46, 0.5] | [0.46, 0.48] |
| LVPW, d [mm] | 0.47 | 0.46 | 0.48 | 0.48 |
|  | [0.45, 0.48] | [0.45, 0.48] | [0.47, 0.5] | [0.46, 0.5] |
| LVID, s [mm] | 0.89 | 0.91 | 1,08 | 1,34 |
|  | [0.78, 1.13] | [0.84, 1.16] | [0.98, 1.27] | [1.14, 1.49] |
| LVID, d [mm] | 2.3 | 2.26 | 2.51 | 2.42 |
|  | [2.23, 2.5] | [2.1, 2.39] | [2.44, 2.69] | [2.34, 2.6] |
| Fractional shortening [%] | 62.07 | 56.39 | 57.9 | 47.56 |
|  | [55.56, 64.23] | [51.78, 59.7] | [51.41, 59.84] | [41.17, 54.52] |
| Ejection fraction [%] | 91.97 | 88.5 | 89.3 | 80.64 |
|  | [87.61, 93.33] | [84.05, 90.82] | [84.58, 90.56] | [74.01, 86.73] |
| Stroke volume [µl] | 16,7 | 15,04 | 19,7 | 16.81 |
|  | [14.85, 17.75] | [13.63, 16.3] | [18.57, 22.82] | [16.27, 19.38] |
| Cardiac output [ml/min] | 12,306 | 8,597 | 14,045 | 10,303 |
|  | [11.152, 13.387] | [8.017, 9.354] | [13.022, 15.623] | [9.476, 11.012] |
| Left ventricular mass [mg] | 22.74 | 21.49 | 44740 | 25.72 |
|  | [21.71, 26.48] | [19.16, 24.16] | [25.54, 30.72] | [23.8, 26.81] |

**Supplemental table S2**. Transthoracic echocardiogram (TTE) data at 7 weeks of age. Data shown as median with confidence intervals (25/75%) in brackets. Abbreviations used: bpm, beats per minute; IVS, interventricular septum; LVPW, left ventricular posterior wall; LVID, left ventricular inner dimension; s, systolic; d, diastolic.

|  | Female | | Male | |
| --- | --- | --- | --- | --- |
|  | **C57BL/6N** | ***Uqcrh*-KO** | **C57BL/6N** | ***Uqcrh*-KO** |
|  | *n*=15 | *n*=15 | *n*=15 | *n*=15 |
|  | median | median | median | median |
|  | [25%, 75%] | [25%, 75%] | [25%, 75%] | [25%, 75%] |
| Heart rate [bpm] | 762.71 | 566.25 | 760.65 | 584.62 |
|  | [745.14, 782] | [550.62, 589.19] | [735.51, 779.55] | [566.04, 611.04] |
| Respiration rate [1/min] | 269.66 | 258.06 | 263.74 | 224.3 |
|  | [237.62, 296.3] | [213.44, 269.7] | [240.6, 280.71] | [190.67, 248.71] |
| IVS, s [mm] | 0.47 | 0.5 | 0.5 | 0.47 |
|  | [0.46, 0.5] | [0.46, 0.5] | [0.48, 0.51] | [0.46, 0.49] |
| IVS, d [mm] | 0.49 | 0.48 | 0.48 | 0.46 |
|  | [0.46, 0.5] | [0.45, 0.48] | [0.47, 0.5] | [0.45, 0.47] |
| LVPW, s [mm] | 0.49 | 0.49 | 0.48 | 0.48 |
|  | [0.48, 0.5] | [0.48, 0.5] | [0.48, 0.5] | [0.47, 0.49] |
| LVPW, d [mm] | 0.49 | 0.48 | 0.47 | 0.49 |
|  | [0.48, 0.5] | [0.47, 0.5] | [0.47, 0.48] | [0.46, 0.5] |
| LVID, s [mm] | 0.98 | 1,13 | 1,09 | 1,4 |
|  | [0.88, 1.15] | [1.04, 1.31] | [0.88, 1.14] | [1.25, 1.5] |
| LVID, d [mm] | 2.5 | 2.33 | 2.47 | 2.54 |
|  | [2.25, 2.65] | [2.19, 2.54] | [2.42, 2.59] | [2.43, 2.57] |
| Fractional shortening [%] | 59.02 | 50.81 | 60.52 | 46.43 |
|  | [57.58, 61.06] | [46.07, 54.48] | [56.96, 62.96] | [40.58, 52.82] |
| Ejection fraction [%] | 90.09 | 83.80 | 90.71 | 79.82 |
|  | [89.08, 91.09] | [79.64, 87.74] | [88.81, 92.66] | [72.94, 85.87] |
| Stroke volume [µl] | 19.86 | 15.67 | 20.87 | 17.95 |
|  | [15.96, 23.18] | [12.83, 18.53] | [18.7, 22.43] | [17.53, 19.97] |
| Cardiac output [ml/min] | 14769 | 8726 | 15873 | 10725 |
|  | [12.082, 17.494] | [7.445, 10.252] | [13.802, 16.724] | [9.765, 11.276] |
| Left ventricular mass [mg] | 26.66 | 24.29 | 27.52 | 26.32 |
|  | [23.99, 30.53] | [20.48, 27.1] | [26.49, 29.05] | [25.22, 28.12] |

**Supplemental table S3**. Transthoracic echocardiogram (TTE) data at 8 weeks of age. Data shown as median with confidence intervals (25/75%) in brackets. Abbreviations used: bpm, beats per minute; IVS, interventricular septum; LVPW, left ventricular posterior wall; LVID, left ventricular inner dimension; s, systolic; d, diastolic.

|  | Female | | Male | |
| --- | --- | --- | --- | --- |
|  | **C57BL/6N** | ***Uqcrh*-KO** | **C57BL/6N** | ***Uqcrh*-KO** |
|  | *n*=15 | *n*=15 | *n*=15 | *n*=15 |
|  | median | median | median | median |
|  | [25%, 75%] | [25%, 75%] | [25%, 75%] | [25%, 75%] |
| Heart rate [bpm] | 772.66 | 578.51a | 750.94 | 565.65a |
|  | [751.41, 795.73] | [529.48, 600.38] | [719.6, 796.1] | [553.38, 580.13] |
| Respiration rate [1/min] | 266.67 | 214.29 | 260.87 | 203.39 |
|  | [234.15, 274.3] | [206.01, 263.74] | [230.3, 292.68] | [192.78, 285.75] |
| IVS, s [mm] | 0.05 | 0.47 | 0.5 | 0.47 |
|  | [0.47, 0.52] | [0.46, 0.48] | [0.48, 0.52] | [0.47, 0.48] |
| IVS, d [mm] | 0.49 | 0.46 | 0.50 | 0.46 |
|  | [0.47, 0.5] | [0.45, 0.47] | [0.48, 0.52] | [0.45, 0.48] |
| LVPW, s [mm] | 0.49 | 0.48 | 0.50 | 0.48 |
|  | [0.47, 0.5] | [0.46, 0.49] | [0.49, 0.5] | [0.47, 0.5] |
| LVPW, d [mm] | 0.48 | 0.50 | 0.50 | 0.50 |
|  | [0.46, 0.5] | [0.48, 0.51] | [0.48, 0.51] | [0.47, 0.52] |
| LVID, s [mm] | 0.91 | 1,06 | 1,12 | 1,25 |
|  | [0.84, 1.08] | [0.96, 1.17] | [0.92, 1.26] | [1.02, 1.48] |
| LVID, d [mm] | 2.43 | 2.13 | 2.58 | 2.38 |
|  | [2.3, 2.65] | [2.12, 2.27] | [2.29, 2.73] | [2.15, 2.5] |
| Fractional shortening [%] | 61.58 | 51.34 | 56.23 | 46.66 |
|  | [57.43, 64.66] | [46.11, 53.4] | [52.56, 59.78] | [39.85, 52.66] |
| Ejection fraction [%] | 91.8 | 83.44 | 88.22 | 80.20 |
|  | [89.03, 93.46] | [79.86, 86.06] | [85.41, 90.42] | [72.54, 85.18] |
| Stroke volume [µl] | 18.43 | 28095 | 19.95 | 15.90 |
|  | [16.25, 22.71] | [11.97, 14.64] | [16.86, 23.82] | [13.69, 18.12] |
| Cardiac output [ml/min] | 13907 | 7436 | 16 | 8523 |
|  | [12.632, 17.339] | [6.908, 8.056] | [12.369, 17.878] | [7.732, 10.047] |
| Left ventricular mass [mg] | 26.73 | 44763 | 30.61 | 25.35 |
|  | [23.59, 29.72] | [20.49, 21.65] | [24.43, 31.9] | [20.84, 27.84] |

**Supplemental table S4**. Transthoracic echocardiogram (TTE) data at 9 weeks of age. Data shown as median with confidence intervals (25/75%) in brackets. Abbreviations used: bpm, beats per minute; IVS, interventricular septum; LVPW, left ventricular posterior wall; LVID, left ventricular inner dimension; s, systolic; d, diastolic.

|  | Female | | Male | |
| --- | --- | --- | --- | --- |
|  | **C57BL/6N** | ***Uqcrh*-KO** | **C57BL/6N** | ***Uqcrh*-KO** |
|  | *n*=15 | *n*=15 | *n*=15 | *n*=15 |
|  | median | median | median | median |
|  | [25%, 75%] | [25%, 75%] | [25%, 75%] | [25%, 75%] |
| Heart rate [bpm] | 750.63 | 551.34 | 759.81 | 552.15 |
|  | [722.02, 754.67] | [505.08, 576.76] | [715.87, 778.78] | [506.8, 566.92] |
| Respiration rate [1/min] | 263.74 | 210.53 | 269.66 | 230.77 |
|  | [245.31, 284.51] | [189.73, 235.38] | [251.31, 313] | [206.19, 289.54] |
| IVS, s [mm] | 0.51 | 0.47 | 0.5 | 0.48 |
|  | [0.48, 0.53] | [0.46, 0.47] | [0.48, 0.53] | [0.46, 0.51] |
| IVS, d [mm] | 0.5 | 0.47 | 0.52 | 0.47 |
|  | [0.48, 0.51] | [0.45, 0.5] | [0.5, 0.54] | [0.44, 0.48] |
| LVPW, s [mm] | 0.5 | 0.47 | 0.52 | 0.49 |
|  | [0.48, 0.5] | [0.47, 0.48] | [0.5, 0.53] | [0.47, 0.5] |
| LVPW, d [mm] | 0.5 | 0.49 | 0.5 | 0.49 |
|  | [0.5, 0.52] | [0.48, 0.5] | [0.48, 0.5] | [0.47, 0.5] |
| LVID, s [mm] | 0.93 | 1,15 | 1,18 | 1,27 |
|  | [0.88, 0.99] | [0.91, 1.57] | [1.05, 1.36] | [1.13, 1.45] |
| LVID, d [mm] | 2.54 | 2.29 | 2.61 | 2.4 |
|  | [2.23, 2.67] | [2.06, 2.73] | [2.54, 3.04] | [2.29, 2.53] |
| Fractional shortening [%] | 62.4 | 49.03 | 56.05 | 49.96 |
|  | [60.09, 64.47] | [40.88, 56.65] | [52.66, 58.85] | [42.48, 51.45] |
| Ejection fraction [%] | 92.21 | 82.14 | 87.97 | 83.21 |
|  | [90.88, 93.08] | [73.68, 88.91] | [85.31, 89.73] | [75.23, 84.62] |
| Stroke volume [µl] | 18.88 | 15,05 | 22.21 | 16.27 |
|  | [15.4, 24.68] | [12.29, 19.38] | [20.34, 29.93] | [12.88, 19.93] |
| Cardiac output [ml/min] | 14259 | 8796 | 17625 | 8984 |
|  | [12.036, 17.818] | [7.064, 9.391] | [15.377, 21.814] | [7.045, 10.008] |
| Left ventricular mass [mg] | 28,02 | 22,5 | 31.86 | 25.64 |
|  | [24.86, 31.57] | [20.57, 31.84] | [29.68, 39.67] | [22.07, 28.75] |

**References**

Gnaiger E (2020) Mitochondrial pathways and respiratory control - An introduction to OXPHOS analysis. 5^th^ ed. Bioenerg Commun 2020.2. <https://doi.org/10.26124/bec:2020-0002>

Gnaiger E, et al - MitoEAGLE-Task-Group (2020) Mitochondrial physiology. Bioenerg Commun 2020.1. <https://doi.org/10.26124/bec:2020-0001.v1>

Lemieux H, Blier PU, Gnaiger E (2017) Remodeling pathway control of mitochondrial respiratory capacity by temperature in mouse heart: electron flow through the Q-junction in permeabilized fibers. Sci Rep-uk 7:2840. <https://doi.org/10.1038/s41598-017-02789-8>
